# H-rev107 regulates prostaglandin D2 synthase-mediated suppression of cellular invasion in testicular cancer cells

**DOI:** 10.1186/1423-0127-20-30

**Published:** 2013-05-20

**Authors:** Rong-Yaun Shyu, Chang-Chieh Wu, Chun-Hua Wang, Tzung-Chieh Tsai, Lu-Kai Wang, Mao-Liang Chen, Shun-Yuan Jiang, Fu-Ming Tsai

**Affiliations:** 1 Department of Internal Medicine, Buddhist Tzu Chi General Hospital Taipei Branch, New Taipei City, Taiwan; 2 School of Medicine, Tzu Chi University, Hualien, Taiwan; 3 Department of Surgery, Tri-Service General Hospital, National Defense Medical Center, Taipei, Taiwan; 4 Department of Dermatology, Buddhist Tzu Chi General Hospital Taipei Branch, New Taipei City, Taiwan; 5 Department of Microbiology, Immunology and Biopharmaceuticals, National Chiayi University, Chiayi, Taiwan; 6 Graduate Institute of Life Sciences, National Defense Medical Center, Taipei, Taiwan; 7 Department of Research, Buddhist Tzu Chi General Hospital, Taipei Branch, New Taipei City, Taiwan

**Keywords:** Murine H-rev107, Human H-REV107, Retinoid-inducible gene 1, Prostaglandin D2 synthase, Testis, HREV107 type II tumor suppressor

## Abstract

**Background:**

H-rev107 is a member of the HREV107 type II tumor suppressor gene family which includes H-REV107, RIG1, and HRASLS. H-REV107 has been shown to express at high levels in differentiated tissues of post-meiotic testicular germ cells. Prostaglandin D2 (PGD2) is conjectured to induce SRY-related high-mobility group box 9 (SOX9) expression and subsequent Sertoli cell differentiation. To date, the function of H-rev107 in differentiated testicular cells has not been well defined.

**Results:**

In the study, we found that H-rev107 was co-localized with prostaglandin D2 synthase (PTGDS) and enhanced the activity of PTGDS, resulting in increase of PGD2 production in testis cells. Furthermore, when H-rev107 was expressed in human NT2/D1 testicular cancer cells, cell migration and invasion were inhibited. Also, silencing of PTGDS would reduce H-rev107-mediated increase in PGD2, cAMP, and SOX9. Silencing of PTGDS or SOX9 also alleviated H-rev107-mediated suppression of cell migration and invasion.

**Conclusions:**

These results revealed that H-rev107, through PTGDS, suppressed cell migration and invasion. Our data suggest that the PGD2-cAMP-SOX9 signal pathway might play an important role in H-rev107-mediated cancer cell invasion in testes.

## Background

H-rev107 [1], also called HRASLS3 [2] or PLA2G16 [3], is a member of the HREV107 type II tumor suppressor gene family, which includes H-REV107, retinoid-inducible gene 1 (RIG1) [4], HRASLS2 [5,6], HRLP5 [7], and HRASLS [8]. The protein in this family contains an NC domain, with unknown function at the N-terminus, and a hydrophobic membrane-anchoring domain at the C-terminus [9,10]. The family proteins exhibit activities that regulate cellular growth, differentiation, and apoptosis, and the membrane-anchoring domain is indispensable for this activity [11-14].

Human H-REV107 and RIG1 have been shown to be involved in the regulation of cellular growth, apoptosis, and differentiation. RIG1 is expressed in highly differentiated tissue derived from skin and colon [13,15,16]. H-REV107 is expressed at high levels in differentiated tissues of post-meiotic testicular germ cells but not in testicular germ cell tumors [17]. Both genes are expressed in normal tissues in a tissue-specific manner and are downregulated in various cancer tissues [15-18]. These proteins exhibit growth-suppressive activities when ectopically expressed in various types of cancer cells and RAS-transformed fibroblasts [6,8,11,13,19-22]. In addition, terminal differentiation of keratinocytes has been observed in cells with induced RIG1 expression [13,23]. Therefore, the HREV107 protein family might play an important role in the regulation of cell growth and differentiation in both normal and cancer cells.

Several studies have observed anti-RAS, phospholipid-metabolizing, and enhancing transglutaminase activities among the HREV107 protein family. Murine H-rev107 was first isolated from revertants of HRAS-transformed fibroblasts [19]. Also, H-REV107 and HRASLS were shown to inhibit the RAS-mediated transformation of fibroblasts [8,20]. Similar inhibition of the RAS signal pathways has been observed in HRASLS2-expressing [6] or RIG1-expressing cervical and gastric cancer cells [21]. The results of our studies further demonstrated a downregulation of activated RAS and total RAS by RIG1 through the post-translational mechanism [11,24]. In addition to the inhibition of RAS, the HREV107 family proteins are phospholipid-metabolizing enzymes. H-REV107 catalyzes the efficient release of free fatty acids and lysophospholipid from phosphatidylcholine, indicating that it acts as phospholipase A [3]. Also, different HREV107 family members catalyze particular phosphatidylcholines or phosphatidylethanolamines [5,7]. In keratinocytes, RIG1 has been shown to stimulate cellular differentiation which is mediated by activating type I tissue transglutaminase [13,23]. These results suggest that RIG1, HRASLS2, and H-REV107 can regulate cellular differentiation in various tissues through different downstream signal pathways.

Prostaglandin D2 (PGD2), which is synthesized by prostaglandin D2 synthase (PTGDS) in many organs, has been implicated as a signaling molecule in the mediation or regulation of various biological processes. PGD2 is expressed in male mice in the early stages of gonadogenesis [25]. Also, PGD2 is shown to contribute to SRY-related high-mobility group box 9 (SOX9) nucleus translocation, which is a critical step of the Sry-initiated testis-determining cascade [26]. The expression of PTGDS is expressed in the cellular lineage that gives rise to the Sertoli cells [27], and the Sertoli cells express PTGDS only during the VI-VIII stages of the spermatogenic cycle, immediately after spermiation [28]. The studies support the role of the PGD2/ PTGDS signaling pathway in the regulation of testis tissue differentiation.

The transcriptional factor SOX9 is involved in cell differentiation, growth and invasion. SOX9 is a master regulator of Sertoli cell differentiation during testis development and is the crucial gene to determine sex [26,29,30]. SOX9 also plays a role in osteochodrogenenic differentiation [31]. SOX9 expression is upregulated by PGD2, and ectopic SOX9 expression has been shown to suppress growth of ovarian cancer and melanoma cells *in vitro* and/or *in vivo*[32]. However, both aberrant SOX9 expression in carcinoma tissues and elevated SOX9 expression are correlated to disease progression and poor prognosis for hepatocellular carcinoma, gastric cancer and prostate cancer [33-35]. SOX9 protein levels are elevated in invasive human uroepithelial carcinoma tissues, which are induced by the activation of epidermal growth factor receptor [34]. Therefore, biological activities of SOX9 appear to be target site specific.

Although high levels of H-REV107 expression in differentiated testes suggest a role of H-REV107 in tissue differentiation, the genuine signaling pathway involved in the H-REV107-mediated cell differentiation of testes remains poorly understood. Our recent study revealed that RIG1 interacted with PTGDS and stimulated PTGDS activity in human testicular NT2/D1 cells [36]. RIG1 is expressed in the human species only, whereas H-REV107 is expressed in both human and murine and exhibits high expression levels in human testes [17]. Since H-rev107 and RIG1 belong to the same protein family, we postulated that the binding of RIG1 to PTGDS might exist between H-rev107 and PTGDS. The present results confirmed the binding between H-rev107 and PTGDS, and demonstrated that the H-rev107-mediated suppression of cell invasion was mediated through the enhancement of PTGDS activity in the murine in testes. Our results suggest that the PGD2 pathway might play an important role in the regulation of H-REV107-mediated testis cell differentiation.

## Methods

### Construction of expression vectors

The H-rev107 and PTGDS cDNA fragments were amplified from mouse TM4 testis cancer cells (Bioresource Collection and Research Center [BCRC], Hsinchu, Taiwan) using H-rev107-specific primers (sense, 5’-TCCTCGAGCTATGCTAGCACCCATACCAGAACCC-3’ and antisense, 5’-TCGGATCCTTGCTTCTGTTTCTTGTTTCTGGAGAGCATG-3’) and PTGDS-specific primers (sense, 5’-TCAAGCTTCGATGGCTGCTCTTCGCATGCTGTG-3’ and antisense, 5’- TCGGATCCGCTCTTGAATGCACTTATCCGGTTGG -3’). To generate pH-rev107-myc and pDsRed-H-rev107, the amplified H-rev107 cDNA fragment was digested with *Xho*I and *Bam*HI and then subcloned in-frame into the multicloning site of the pcDNA3.1-myc-his A expression vector (Invitrogen, Carlsbad, CA, USA) or pDsRed-C1 (Clontech Laboratories, Inc, Palo Alto, CA, USA). To generate pPTGDS-Flag and pEGFP-PTGDS, the amplified PTGDS cDNA fragment that had been digested with *Hin*dIII-*Bam*HI was cloned in-frame into the pPCR3.1-Flag (Dr. Yi-Ling Lin, Institute of Biomedical Science, Academia Sinica, Taipei, Taiwan) and pEGFP-C1 (Clontech Laboratories). The cDNA sequences of fusion proteins were confirmed by DNA sequencing.

### Immunohistochemical analysis

Testes tissue sections from Balb/c mice were deparaffinized with trilogy (Cell Marque, Rocklin, CA, USA) and rehydrated in a graded series of ethanol. To retrieve antigens, the sections were boiled for 30 min in 10% DAKO Chem-Mate™ solution (DAKO Co., Carpinteria, CA, USA) containing 0.05% Nonidet P-40. Endogenous peroxidase activity was blocked by incubation in 3% hydrogen peroxide for 10 min. The sections were then incubated at room temperature for 2 h in H-REV107 (Biorbyt, Cambridge, Cambridgeshire, UK), PTGDS (Santa Cruz Biotechnology, Santa Cruz, CA, USA), or control rabbit IgG antibody (Santa Cruz Biotechnology) diluted at 1:1000, 1:200, or 1:400 respectively in DAKO antibody diluent. The DAKO LSAB^®^ 2 Peroxidase kit was used to stain protein expression in tissue sections. Sections were incubated with 3-3’-diaminobenzidine chromogen solution (DAKO Co) for 5 min to reveal the peroxidase complex. Finally, sections were lightly counterstained with Mayer’s hematoxylin (Merck, Darmstadt, Germany) and mounted with DPX mounting medium (Schrlau, Spain). Our study had been reviewed and approved by the Buddhist Tzu Chi General Hospital-Taipei Branch Institutional Animal Care and Use Committee.

### Cell culture and transfection

Mouse TM3 mouse Leydig and TM4 mouse Sertoli cells (BCRC) were maintained in a growth medium consisting of a 1:1 mixture of Ham’s F12 medium and Dulbecco's Modified Essential Medium (DMEM) supplemented with 4.5 g/L glucose, 2.5 mM L-glutamine, 0.5 mM sodium pyruvate, 1.2 g/L sodium bicarbonate, 15 mM HEPES, 5% horse serum, and 2.5% fetal bovine serum (FBS). Human NT2/D1 teratocarcinoma cancer cells were maintained in DMEM supplemented with 25 mM HEPES, 26 mM NaHCO_3_, 2 mM L-glutamine, penicillin (100 units/mL), streptomycin (100 μg/mL), and 10% FBS at 37°C in 5% CO_2_. Cells plated in culture dishes were transfected with the expression vectors using liposome-mediated transfection. Plasmids and lipofectamine 2000 (Gibco BRL, Gaithersburg, MD, USA) were diluted in Opti-MEM medium and then mixed with plasmids at room temperature for 15 min. The DNA–lipofectamine complexes were then added to cells for 5 h at 37°C. Cells were refreshed with complete medium for 24 h at 37°C for further analysis.

### Immunoprecipitation and Western blotting

Cells were lysed in IP lysis buffer (20 mM Tris-HCl at pH 7.5, 100 mM NaCl, 1% Nonidet P40, 100 μM Na_3_VO_4_, 50 mM NaF, and 30 mM sodium pyrophosphate) containing 1× complete protease inhibitor cocktail (EDTA-free) (Roche Diagnostics, Mannheim, Germany). Cell lysates containing 500 μg of protein were first incubated first with 3.2 μg of anti-myc (Invitrogen) or 1 μg of anti-Flag-M2 (Sigma, St. Louis, MO, USA) monoclonal antibody for 2 h at 4°C and then incubated with 20 μL of protein G plus /protein A agarose (Calbiochem, Cambridge, MA, USA) at 4°C for 2 h. Immunoprecipitated complexes were washed three times with IP lysis buffer and then analyzed by Western blotting using an anti-myc or anti-Flag antibody. For Western blotting, proteins (20–50 μg) were separated on 12% polyacrylamide gels and transferred to polyvinylidene difluoride membranes. After blocking, membranes were incubated with anti-myc, anti-Flag, anti-PTGDS (Abcam, Cambridge, UK), anti-phospho-SOX9 (Abcam), anti-SOX9 (Abcam), anti-E-cadherin (Santa Cruz Biotechnology, Santa Cruz, CA, USA), anti-vimentin (Santa Cruz Biotechnology), or anti-actin (Sigma) antibody for 12 h at 4°C and then incubated with horseradish peroxidase-conjugated goat anti-mouse or anti-rabbit antibody at room temperature for 1 h. An ECL kit (Amersham, Bucks,UK) was used to detect the substrate reaction.

### Confocal and immunofluorescent analysis

TM4 cells (1 × 10^5^) were plated on poly-L-lysine-coated coverslips in 35-mm dishes in growth medium. Cells were then transfected with 500 ng of DsRed-H-rev107 along with 500 ng pEGFP-PTGDS expression vector for 18 h. The cells were washed, fixed with 4% paraformaldehyde, stained with 1 μg/mL 4’6-diamidino-2-phenylindole (DAPI), and then analyzed with a Leica TCS SP5 scanner (Leica, Bensheim, Germany). The fluorescent images were then processed with Image-Pro Plus 5.1 image analysis software.

### Measurement of PGD2 and cAMP levels

Cells were cultured onto 6-well plates overnight and then transfected with 500 ng of pPTGDS-Flag along with 500 ng of pH-rev107-myc, or control vector in complete medium for 5 h. Cells were incubated in complete medium supplemented with 1 mM Br-cAMP or ethanol vehicle for 18 h. Alternatively, cells were washed and then incubated with 1 μg/mL arachidonic acid (AA, Sigma) for 1 h or PGD2 (500 ng/mL) for 30 min immediately before harvest. After washing twice with PBS, cells were lysed with 0.1 N HCl for 20 min, scraped, and collected by centrifugation. Levels of PGD2 or cAMP in the supernatants were determined using a prostaglandin D2 express or cyclic AMP EIA kit (Cayman Chemical, Ann Arbor, MI, USA) according to the manufacturer's instructions.

### Cell migration and invasion assay

For cell migration assay, NT2/D1 cells (2 × 10^4^) were added to the upper polycarbonate membrane insert (8 μm pore size; Falcon, Becton, Dickinson and Company, Franklin Lakes, NJ, USA) of the cell migration assay kit in a 24-well plate. In the lower well, seven hundred μL of DMEM supplemented with 20% FBS was used as chemoattractant. After 24 h of incubation, cells were methanol fixed for 10 min at room temperature and then stained for 30 min at room temperature with a 50 μg/mL solution of propidium iodide (Sigma).

Polycarbonate-membrane inserts coated with 30 μg Matrigel (BD) were used for cell invasion assays. NT2/D1 cells (2 × 10^4^), suspended in DMEM medium containing 10% NuSerum (BD), were seeded in the membrane insert. Seven hundred μL of serum-containing medium supplemented with PGD2 (500 ng/mL) or ethanol vehicle was placed in the lower chambers, with the medium changed daily for 72 h at 37°C. Cells were fixed and stained for propidium iodide. The number of cells on each membrane was counted under a microscope at a magnification of 40×. Experiments were performed at least twice, and each sample was assayed in triplicate.

### Viruses and transduction

LacZ, PTGDS, and SOX9-shRNA-containing lentiviral vectors were obtained from the National RNAi Core Facility (Academia Sinica, Taiwan) and prepared in accordance with standard protocols. Cells were infected with lentivirus (multiplicity of infection 5) in medium containing polybrene (8 μg/mL). Two PTGDS shRNAs targeted to nucleotides 540 to 560 (5’-CAGGGCTGAGTTAAAGGAGAA-3’) and 625 to 645 (5’-GATAAGTGCATGACGGAACAA-3’) were synthesized based on Genbank accession NM_000954. Two SOX9 shRNAs targeted to nucleotides 1761 to 1781(5’-GATAAGTGCATGACGGAACAA-3’) and 3680 to 3700 (5’-GCATCCTTCAATTTCTGTATA-3’) were synthesized based on Genbank accession NM_000346.

### Rac activation assays

Cells grown to 80% confluence in 10-cm culture dishes were first transfected with 5 μg H-rev107 or control expression vector and then incubated with 500 ng/mL of PGD2 or ethanol vehicle for 24 h. Cells were serum starved for 12 h and then stimulated with 50 ng/mL epidermal growth factor (EGF, Sigma) for 5 min at 37°C. Rac1 activity was assessed using the Rac1 activation assay kit (Millipore, Temecula, CA, USA). Briefly, cells were washed twice with ice-cold PBS and then lysed in 0.5 mL MLB buffer (25 mM HEPES, pH 7.5, 150 mM NaCl, 1% Igepal CA-630, 10 mM MgCl_2_, 1 mM EDTA, and 10% glycerol) containing protease inhibitors and phosphatase inhibitors. Cellular lysates containing 300 μg protein were then incubated with 10 μL of the PAK-1 PBD agarose bound with glutathione S-transferase fusion protein corresponding to the human p21 binding domain (PBD, residues 67–150) of human PAK-1 at 4°C for 1 h. After washing three times with MLB containing protease and phosphatase inhibitors, presence of the activated Rac1 (Rac1-GTP) was detected by Western blotting using an anti-Rac1 monoclonal antibody (Millipore).

## Results

### Expression of H-rev107 and PTGDS in mouse testes

**Figure 1:**
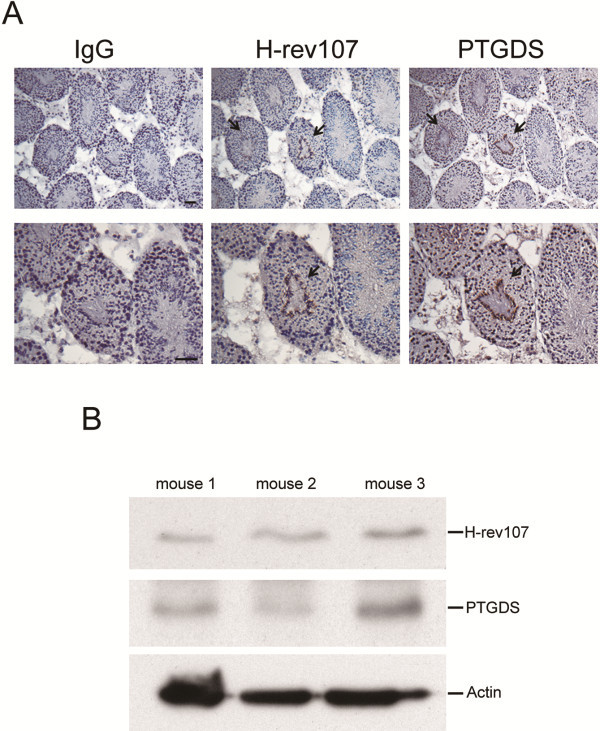
**Expression of H-rev107 and PTGDS in normal testis tissues.** Mouse testis tissues were analyzed for expression of H-rev107 or PTGDS by immunohistochemical staining using rabbit IgG, H-rev107, or PTGDS antibody. Arrows indicate positive H-rev107 and PTGDS staining (magnification ×100, top panel; ×200, bottom panel) (**A**). Total cellular extracts from testis of mice were subjected to Western blot analysis for H-rev107, PTGDS, and actin (**B**). Scale bar: 50 μm.

To analyze the expression of H-rev107 and PTGDS proteins in the testis of Balb/c mice, we performed an immunohistochemical analysis. Strong H-rev107 expression was detected in spermatids, and no H-rev107 expression was observed in spermatogonia and spermatocytes (Figure 1A). Localization of H-rev107 protein was similar to H-REV107 RNA detected in human testis [17]. Similarly, positive PTGDS staining was observed only in spermatids (Figure 1A). Therefore, H-rev107 and PTGDS were both expressed in the terminally differentiated testis tissues. No staining was observed in tissues incubated with rabbit control IgG. The expression of H-rev107 and PTGDS was also confirmed by Western blotting in testis cell extracts prepared from three mice (Figure 1B).

### H-rev107 associates and co-localizes with PTGDS

**Figure 2:**
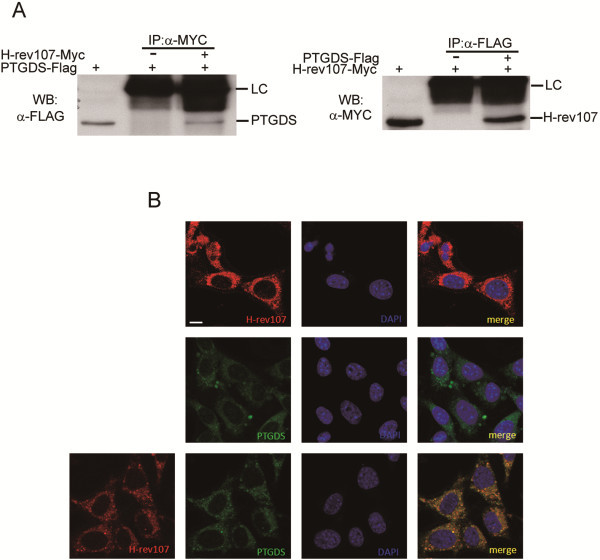
**H-rev107 associates and co-localizes with PTGDS.** Mouse TM4 cells plated in a 10-cm dish were transfected with 3 μg PTGDS-Flag along with H-rev107-myc or the control vector for 24 h. Cell lysates were prepared as described in the Methods. The interaction between H-rev107 and PTGDS was analyzed by immunoprecipitation followed by Western blot analysis. Immunoprecipitates were resolved by SDS-PAGE and immunoblotted using anti-Flag antibody or anti-myc antibody (**A**). TM4 cells were transfected with EGFP-PTGDS or DsRED-H-rev107 expression vector alone or co-transfected with both vectors for 18 h. Cells were fixed, stained with DAPI, and analyzed with a laser scanning confocal microscope (**B**). LC: light chain; scale bar: 10 μm.

RIG1 can interact with PTGDS [36]. Whether H-rev107 is associated with PTGDS was evaluated. Myc-tagged H-rev107 or Flag-tagged PTGDS fusion proteins with expected molecular weights of 22.7 and 23.9 kDa, respectively, were detected in cytosol extracts prepared from transfected TM3 and TM4 cells (data not shown). We further performed immunoprecipitation using anti-myc antibody specific to the myc epitope of the H-rev107 fusion protein in lysates of PTGDS-Flag and H-rev107-Myc co-transfected TM4 cells. In Figure 2A, the left panel shows that PTGDS coprecipitated with H-rev107 in TM4 cell lysates. Similarly, H-rev107 was observed on immunoblots from PTGDS co-transfected immunoprecipitates (Figure 2A, right panel). A similar interaction between H-rev107 and PTGDS was observed in TM3 cells (data not shown). We next verified the co-localization between H-rev107 and PTGDS within cells. DsRed-H-rev107 and EGFP-PTGDS expression vectors were co-transfected into TM4 cells for 18 h. Both DsRed-H-rev107 and EGFP-PTGDS were expressed in cytoplasm with preferential localization at the peri-nuclear region (Figure 2B), and most of the DsRed-H-rev107 and EGFP-PTGDS proteins were co-localized (yellow) in co-transfected cells (Figure 2B and Additional file 1: Figure S1).

### H-rev107 enhances PTGDS activity in human NT2/D1 testis cancer cells

**Figure 3:**
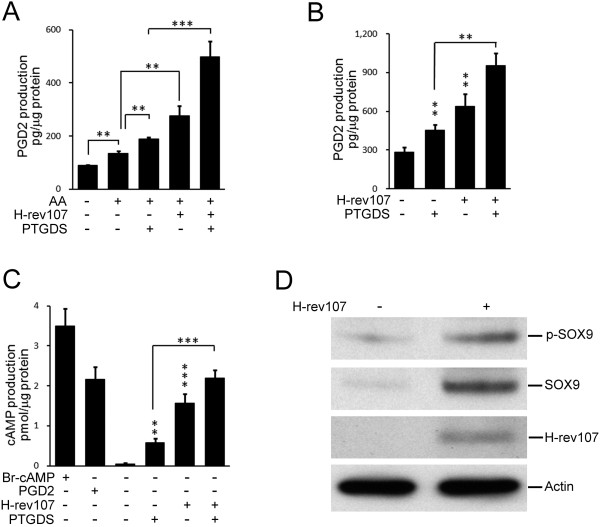
**H-rev107 increases PGD2 and cAMP levels and enhances the expression of SOX9 protein.** NT2/D1 cells plated in triplicate in 6-well plates were transfected with 1 μg of indicated expression or control vector for 24 h. Cells were washed and then incubated with 1 μg/mL AA for 1 h (**A**). Alternatively, cells were transfected with indicated expression or control vector and then incubated for 18 h in the presence of 1 mM Br-cAMP or ethanol vehicle for 18 h or 500 ng/mL PGD2 for 30 min as described in the Methods. Cell lysates were prepared, and levels of PGD2 (**A**, **B**) or cAMP (**C**) were measured using an enzyme immunoassay. Representative results from triplicate samples were expressed as mean ± SD. Student’s *t*-test: **, *P* <0.01; ***, *P* < 0.001. Cells were transfected with H-rev107 expression or control vector for 24 h. Expression of SOX9 and phosphorylated SOX9 in total cellular extracts was determined by Western blot analysis (**D**).

The PGD2-SOX9 pathway has been well-studied in human NT2/D1 teratocarcinoma cells [26]. To examine the effect of H-rev107 on PTGDS activity, NT2/D1 cells were co-transfected with the PTGDS expression vector and either H-rev107 or a control vector for 24 h. AA treatment significantly increased PGD2 levels by 52% in control transfected cells (Figure 3A). Among AA treated cells, PGD2 levels were increased by 40 or 105% in PTGDS- or H-rev107-transfected cells, respectively. Compared to the PTGDS-transfected cells, the levels of PGD2 were furthered increased by 165% when cells co-expressed PTGDS and H-rev107 (Figure 3A). In the absence of exogenous AA addition, PGD2 levels were increased by 59 or 124% in PTGDS- or H-rev107-transfected cells, respectively (Figure 3B). Compared to PTGDS–expressiong cells, PGD2 levels were enhanced by 234% in PTGDS and H-rev107 co-transfected cells. To dissect the effects of H-rev107 on PGD2 downstream signalling molecules, we first measured the levels of cAMP, a marker for the activation of PTGDS. NT2/D1 cells were transfected with PTGDS expression vector along with H-rev107 or control vector. PGD2 profoundly increased intracellular cAMP by 42.9-fold in control transfected NT2/D1 cells, slightly less than the effects of the constitutive cAMP activator, Br-cAMP (Figure 3C). Levels of cAMP production were increased by 11.6-fold in PTGDS-expressing cells and were further increased by 43.6-fold when the cells co-expressed PTGDS and H-rev107. Expression of H-rev107 alone in NT2/D1 cells also enhanced cAMP levels by 31.2-fold. We also analyzed the downstream signal followed by the activation of PTGDS using Western blotting as shown in our previous study [36]. Levels of total SOX9 as well as the phosphorylated (p-SOX9) proteins were increased by 3.4- and 8.3-fold, respectively, followed by H-rev107 expression in NT2/D1 cells (Figure 3D). These results revealed that H-rev107 can enhance PTGDS activity in NT2/D1 cells.

### H-rev107 suppresses NT2/D1 cell migration and invasion

**Figure 4:**
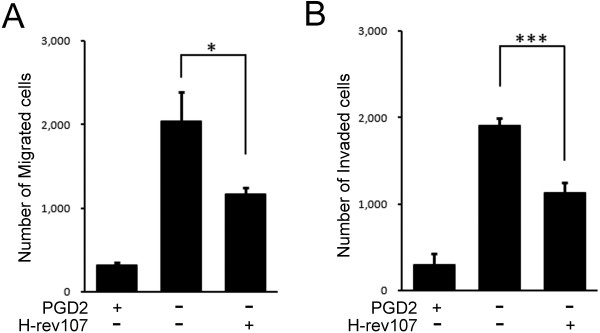
**H-rev107 suppresses cell migration and invasion.** NT2/D1 cells plated in triplicate in 24-well plates were transfected with 500 ng of H-rev107 or control expression vector and then incubated with 500 ng/mL of PGD2 or ethanol vehicle for 24 h. Cells were washed with serum free medium and were seeded in triplicate in polycarbonate membrane inserts in 24-well plates. Lower wells containing 700 μL DMEM supplemented with 20% FBS was served as chemoattractant. Migratory cells were stained after 24 h of incubation (**A**). Invasive activity was measured using the Matrigel invasion assay after 72 h of incubation in serum containing medium supplemented with PGD2 or ethanol vehicle (**B**). Representative results of three independent experiments are shown. Ctrl: control; Student’s *t*-test: *, *P* < 0.05; ***, *P* < 0.001.

Expression of RIG1 in NT2/D1cells has been shown to inhibit cell migration and invasion through the PGD2 signal pathway [36]. This study investigated the effect of H-rev107 on cell migration and invasion in NT2/D1 cells. Numbers of migrated and invaded cells were decreased by 84.4% and 84.7%, respectively followed by PGD2 treatment (Figure 4). Similarly, expression of H-rev107 decreased the numbers of migrating or invaded cells by 42.5% or 59%, respectively. The effect of H-rev107 on cell viability and cell death were also investigated by the WST-1 and LDH assay, respectively, on H-rev107 and control transfected NT2/D1 cells, and no effect was observed (data not shown). These results indicated that H-rev107 and RIG1 exhibited similar inhibitory effects on cell migration and invasion in NT2/D1 teratocarcinoma cells.

### PGD2, cAMP, and SOX9 induction by H-rev107 was mediated through PTGDS

**Figure 5:**
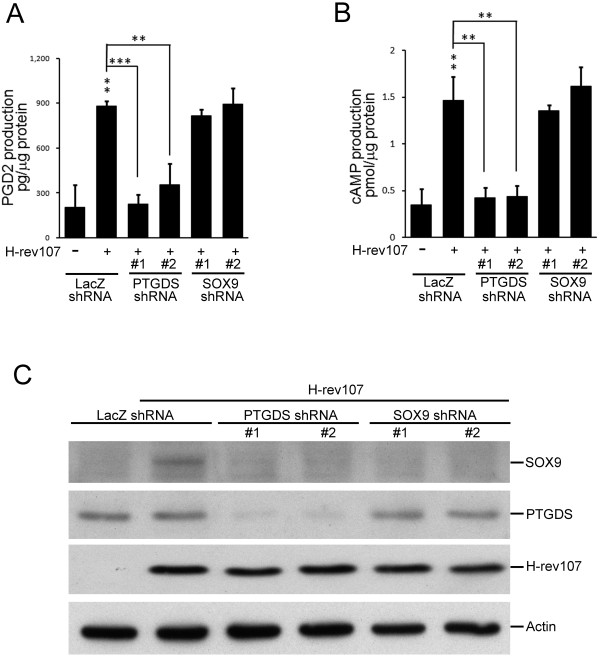
**PTGDS shRNAs prevent H-rev107-mediated enhancement of PGD2, cAMP, and SOX9 levels.** NT2/D1 cells were transduced with indicated shRNA for 72 h and then transfected with H-rev107 expression or control vector for 24 h. Levels of PGD2 (**A**) and cAMP (**B**) were determined using enzyme immunoassays, and expression of SOX9 was determined by Western blot analysis using anti-SOX9 antibody (**C**). Representative results from triplicate samples were expressed as means ± SD. Student’s *t*-test: **, *P* < 0.01; ***, *P* < 0.001.

Given that H-rev107 can induce PTGDS activity in NT2/D1 cells, we investigated whether PTGDS is essential for the H-rev107-mediated PGD2 signal pathway. We first silenced PTGDS and SOX9 expression and then examined the production of PGD2 and cAMP in H-rev107-expressing NT2/D1 cells. Levels of PTGDS and SOX9 proteins were effectively downregulated by the transduction of specific shRNAs (Figure 5C). Levels of PGD2 and cAMP were increased by 3.4- and 3.2-fold respectively in LacZ-transduced and H-rev107-expressed cells. Among H-rev107 transfected cells, silencing of PTGDS decreased PGD2 production by 59.9% to 74.3% (Figure 5A) and decreased cAMP levels by 69.9% to 70.8% (Figure 5B). No effect on the PGD2 or cAMP production was observed in cells transduced with SOX9 shRNA. Furthermore, levels of H-rev107-induced SOX9 expression were lower than that of LacZ-silencing cells when cells were transduced with PTGDS shRNA (Figure 5C). The results suggest that H-rev107 stimulates PGD2 production and downstream signals by enhancing PTGDS activity.

### H-rev107 suppresses cell migration and invasion through PTGDS

**Figure 6:**
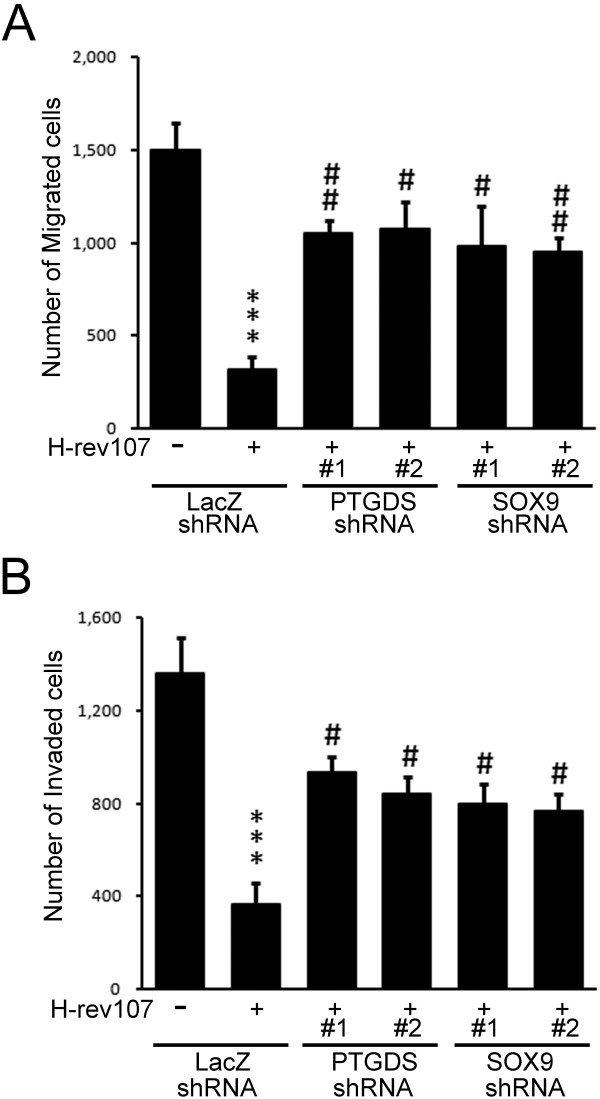
**PTGDS and SOX9 shRNA alleviates H-rev107-mediated suppression of cell migration and invasion.** NT2/D1 cells were transduced with indicated shRNA for 72 h and then transfected with H-rev107 expression vector for 24 h. Cells were then prepared for analysis of cell migration (**A**) and invasion (**B**). Representative results of three independent experiments are shown. Student’s *t*-test: ***, *P* < 0.001 versus control; ^#^, *P* <0.01; ^##^, *P* <0.001 versus H-rev107-expressing cells that transduced with shLacZ.

Having found that expression of H-rev107 will increase PGD2 production by modulating PTGDS activity, we then determined the role of PTGDS and SOX9 in H-rev107-mediated suppression of cell migration and invasion. H-rev107 suppressed cell migration and invasion by 79.1% and 73.4% respectively in the NT2/D1 cells transduced with LacZ shRNA (Figure 6). In contrast, silencing of PTGDS in NT2/D1 cells increased cell migration by 49% to 50.6%, and cell invasion by 40.6% to 47.5% in H-rev107 transfected cells. Similarly, silencing of SOX9 in NT2/D1 cells reversed the H-rev107-mediated suppression of cell migration by 42.3% to 44.6% and cell invasion by 35.5% to 37.5% (Figure 6). Both PTGDS and SOX9 shRNAs significantly alleviated the PGD2 mediated inhibition of cell migration and invasion in NT2/D1 cells (Additional file 2: Figure S2).

### H-rev107 suppresses Rac1 activation and increases E-cadherin expression

**Figure 7:**
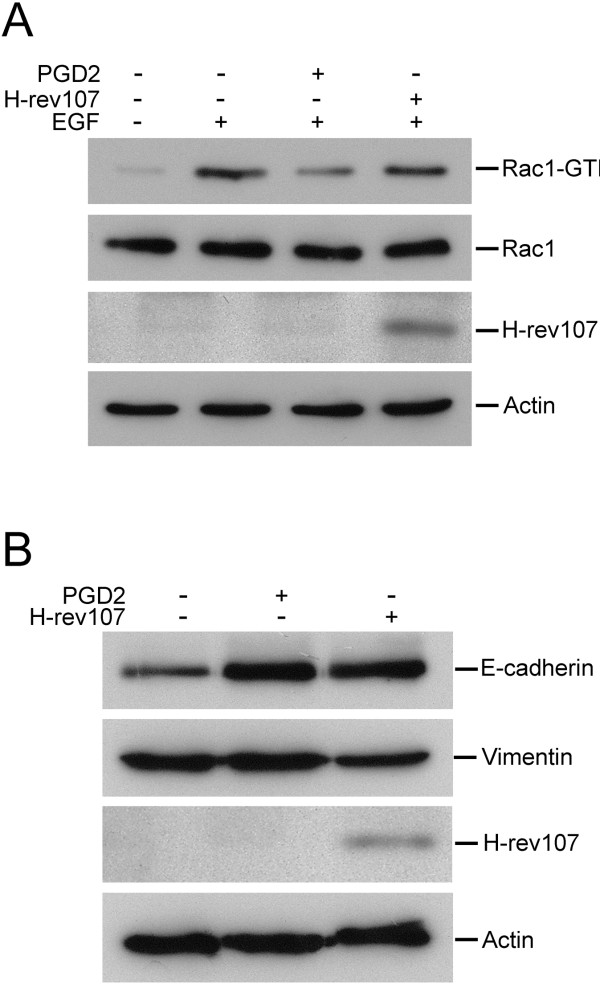
**H-rev107 suppresses Rac1 activation and increases E-cadherin expression.** NT2/D1 cells grown to 80% confluence were transfected with the indicated control or H-rev107 expression vector and then incubated with 500 ng/mL of PGD2 or ethanol vehicle for 24 h. Cells were serum starved for 12 h and then stimulated by EGF (50 ng/mL) for 5 min. Cellular lysates were incubated with agarose conjugated with PAK-1 PBD. After washing, the bound activated Rac1 (Rac1-GTP) was analyzed by Western blotting (**A**). NT2/D1 cells were transfected with H-rev107 or control expression vector and then incubated with 500 ng/mL of PGD2 or ethanol vehicle for 24 h. The levels of E-cadherin and vimentin were determined by Western blot analysis (**B**).

The conversion of an epithelial cell to a mesenchymal cell is necessary for cell migration and invasion [37,38]. We next examined the effects of H-rev107 on the activation of cellular Rac1 and the expression of E-cadherin and vimentin. EGF stimulated Rac1-GTP levels by 14.3-fold in NT2/D1 cells (Figure 7A). Compared to the control transfected cells, the levels of EGF-stimulated Rac1-GTP were suppressed by 62.6 and 21.9% in PGD2-treated or H-rev107-transfected cells, respectively. In addition, PGD2 treatment or H-rev107 transfection increased E-cadherin levels by 1.7-1.9 fold (Figure 7B). Vimentin expression was downregulated to 20% only in H-rev107-transfected cells. PGD2 had no effect on vimentin expression. We also analyzed the effect of PGD2 and H-rev107 on matrix metallopeptidase (MMP) activation. However, no change in the activity of MMP-9 or MMP-2 was observed in NT2/D1 cells treated with PGD2 or transfected with H-rev107 expression vector (data not shown).

## Discussion

Based on the results from the present and our previous [36] studies, both RIG1 and H-rev107 can interact with PTGDS in testis cells. The interaction enhances PTGDS activity, which increases PGD2 levels, elevates or activates downstream PGD2 signaling molecules like cAMP and phosphorylated SOX9, and suppresses cell migration and invasion. Both PTGDS and SOX9 shRNAs profoundly alleviated RIG1-, H-rev107-, and PGD2-mediated inhibition of cell migration and invasion. Therefore, the mechanism by which HREV107 family proteins attenuate the migration and invasion of NT2/D1 cells is primarily mediated through the activation of PTGDS and the production of PGD2.

PGD2 has been shown to inhibit cell migration and invasion. PGD2 inhibits the migration of airway dendric cells and epidermal Langerhans cells to the draining lymph nodes, and the inhibition is mediated through prostanoid receptor 1 [39,40]. Similar inhibition of cell migration by PGD2 is also observed in eosinophils, basophils and lung fibroblasts [41,42]. PGD2 inhibited cell invasion, whereas PGE2 stimulated invasion of PC-3 prostate cancer cells [43]. Also, PGD2 levels in primary colorectal carcinoma tissues without liver metastasis are shown to be significantly lower than that with hepatic metastasis [44]. The results agree with the inhibition of cell migration and invasion in NT2/D1 testis cancer cells followed by PGD2 treatment or the ectopic expression of RIG1 or H-rev107 shown in this and our previous studies [36]. Epithelial-mesenchymal transition and elevated Rac activities have critical roles in cellular motility and migration. PGD2 is shown to inhibit TGF-β1-induced epithelial-mesenchymal transition by increasing E-cadherin in MDCK cells [45]. Similarly, an increase in expression of E-cadherin and a decrease in expression of mesenchymal marker protein vimentin and in Rac 1 activation were observed in NT2/D1 cells that expressed H-rev107. These results confirmed the invasion-suppression capacity of H-rev107 in testes cells. SOX9 is shown to be required in migration and in invasion of uroepithelial carcinoma cells *in vitro*[34], and upregulation of SOX9 is related to the progression of prostate and gastric cancers [33-35]. However, we observed that knockdown of PTGDS or SOX9 expression effectively alleviated both RIG1 [36] and H-rev107-mediated inhibition of cell migration and invasion in testis cancer cells. The difference in the activities of SOX9 in cell migration and invasion might be attributable to the tissue specific effects of the protein.

The PGD2-SOX9 signal pathway is important in testis development [26]. PDG2 induces nuclear import of SOX9 that subsequently induces Sertoli cell differentiation [26]. The facts that the increase in PGD2 production and SOX9 expression through PTGDS activation in H-rev107 and RIG1 transfected NT2/D1 cells shown in this and our previous [36] studies support pro-differentiation activities of both RIG1 and H-rev107 in testis cancer cells. This is consistent with the finding that only terminal differentiated testis tissues appear to contain murine H-rev107, human HREV107 [17] and PTGDS. Results from this and our previous [36] studies demonstrated similar biological activities between RIG1 and H-rev107 in the activation of PTGDS that subsequently increase the level of PGD2 and SOX9 and inhibit cell migration and invasion. Whether the activities described above differ in potency between RIG1 and H-rev107 remains unclear. A side-by-side comparison of RIG1 and H-rev107 expression and downstream signaling pathways will clarify the roles of RIG1 and H-rev107 in testes differentiation and in the inhibition of testis cell invasion.

Previous studies have shown that the HREV107 family proteins exhibit tumor suppressor activities in combination with various target proteins. In cervical cancer, RIG1 suppresses cell growth and induces cell death through caspase-dependent and -independent pathways [12,24]. In skin cancer, RIG1 induces cell apoptosis by promoting pericentrosomal organelle accumulation, which is associated with the decrease in cyclin D1, cyclin E, and Bcl-XL and the increase in p21 and Bax levels [22,46]. In addition, both RIG1 and H-REV107 have been suggested to exhibit phospholipase A(1/2) activity [3,5], which is involved in H-rev107-mediated HEK cell death by regulating peroxisomal lipid metabolism [47]. However, pro-apoptotic activity of H-REV107 has not been observed in testis cells. The use of phospholipase A(1/2) inhibitor cannot alleviate the RIG1-mediated suppression of cell invasion [36]. These results reveal that the targeted effects for the HREV107 family proteins vary by cell type.

Aside from the difference in targeted proteins for H-REV107, subcellular localization of H-REV107 would be considered as an important factor that might have impact on cell function. Nuclear targeted H-REV107 has been shown to stimulate cell growth of non-small cell lung carcinomas [48]. In contrast, nuclear targeted H-REV107_111–123_ and RIG1_111–123_ peptides induce profound proapoptotic activities in cancer cells [12,49]. Results from most studies have revealed that the HREV107 family proteins are expressed in the perinuclear region [6,13,14,23,24]. Perinuclear localization of RIG1 has been shown to inhibit expression or activation of signaling molecules such as HER2, RAS, PI3K/AKT, mTOR, and type I transglutaminase that are involved in the regulation of cell growth, apoptosis, tumor invasion, and cell differentiation [11-13,24,50]. The downstream signal transduction pathways involved in RIG1-mediated cell function are dependent on the cell type and the binding effectors. For example, the transglutaminase inhibitor monodansylcadaverine can suppress RIG1-mediated terminal differentiation of keratinocytes [23]. However, the compound is not able to inhibit RIG1-mediated RAS suppression and induce cell death of cervical cancer cells (data not shown).

Results from this and our previous studies [36] support the roles of RIG1/H-rev107 in testis cell invasion/migration. However, a signal cascade involving RIG1/H-rev107-PTGDS-SOX9 has also been implicated in testis development and differentiation based on results from this and previous [17,26] studies. Due to the lack of sex-differentiation marker like Mullerian hormone and Sertoli cell marker [25] in cell line culture, an organ culture of testis with Sertoli cells that support spermatogenesis at various stages of cell differentiation will be used in our future studies. Also, analysis of H-rev107 in the sex-determining cascade *in ex vivo* using *H*-*rev107* knockout mice will be helpful in identifying the signal responsible for H-rev107-mediated testis development.

## Conclusions

In conclusion, H-rev107 and PTGDS are both highly expressed in differentiated spermatids in normal testis tissues. H-rev107 exhibited invasion-suppressive activity in testis cancer cells. PTGDS is essential for H-rev107-mediated production of PGD2, cAMP, and SOX9. Furthermore, reduction of PTGDS or SOX9 alleviates the H-rev107 mediated suppression of cell migration and invasion. Further analysis of H-rev107 in gene knockout mice will be useful to pinpoint the role of H-rev107 in testis development.

## Abbreviations

DAPI: 4’6-Diamidino-2-phenylindole; DMEM: Dulbecco’s modified essential medium; FBS: Fetal bovine serum; PGD2: Prostaglandin D2; PTGDS: Prostaglandin D2 synthase; RIG1: Retinoid-inducible gene 1; SOX9: SRY-related high-mobility group box 9.

## Competing interests

The authors declare that they have no competing interests.

## Authors’ contribution

R-YS designed research and supervised the experiments; C-CW, C-HW, L-KW, M-LC designed research and data discussion; T-CT and F-M T performed the experiments, contributed to experimental design, and drafted the manuscript; S-YJ supervised the experiments, assisted in the writing of and proofed the manuscript. All authors read and approved the final draft of the manuscript.

## Supplementary Material

Additional file 1: Figure S1 TM4 cells were co-transfected with EGFP-PTGDS and DsRED-H-rev107 expression vector for 18 h. Cells were fixed, stained with DAPI, and analyzed with a laser scanning confocal microscope. Scale bar: 10 μm. Click here for file

Additional file 2: Figure S2 PTGDS and SOX9 shRNAs alleviate PGD2-mediated suppression of cell migration and invasion. NT2/D1 cells were transduced with indicated shRNA for 72 h and then incubated with 500 ng/mL of PGD2 or ethanol vehicle for 24 h. Cells were subsequently prepared for analysis of cell migration (**A**) and invasion (**B**). Representative results of three independent experiments are shown. Student’s *t*-test: **, P < 0.01 versus control; ^#^, P <0.01; ^##^, P <0.001 versus PGD2-treated cells that transduced with shLacZ. Click here for file
