# Supplementary figures and images for: H-rev107 regulates prostaglandin D2 synthase-mediated suppression of cellular invasion in testicular cancer cells
Source: J Biomed Sci. 2013 May 20;20(1):30. doi: 10.1186/1423-0127-20-30 (PMC3669107; doi:10.1186/1423-0127-20-30)

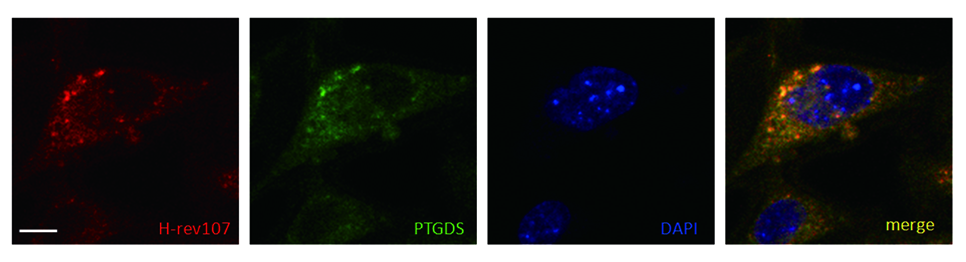

Supplement: Additional file 1: Figure S1 — TM4 cells were co-transfected with EGFP-PTGDS and DsRED-H-rev107 expression vector for 18 h. Cells were fixed, stained with DAPI, and analyzed with a laser scanning confocal microscope. Scale bar: 10 μm. [file 1423-0127-20-30-S1.tiff]

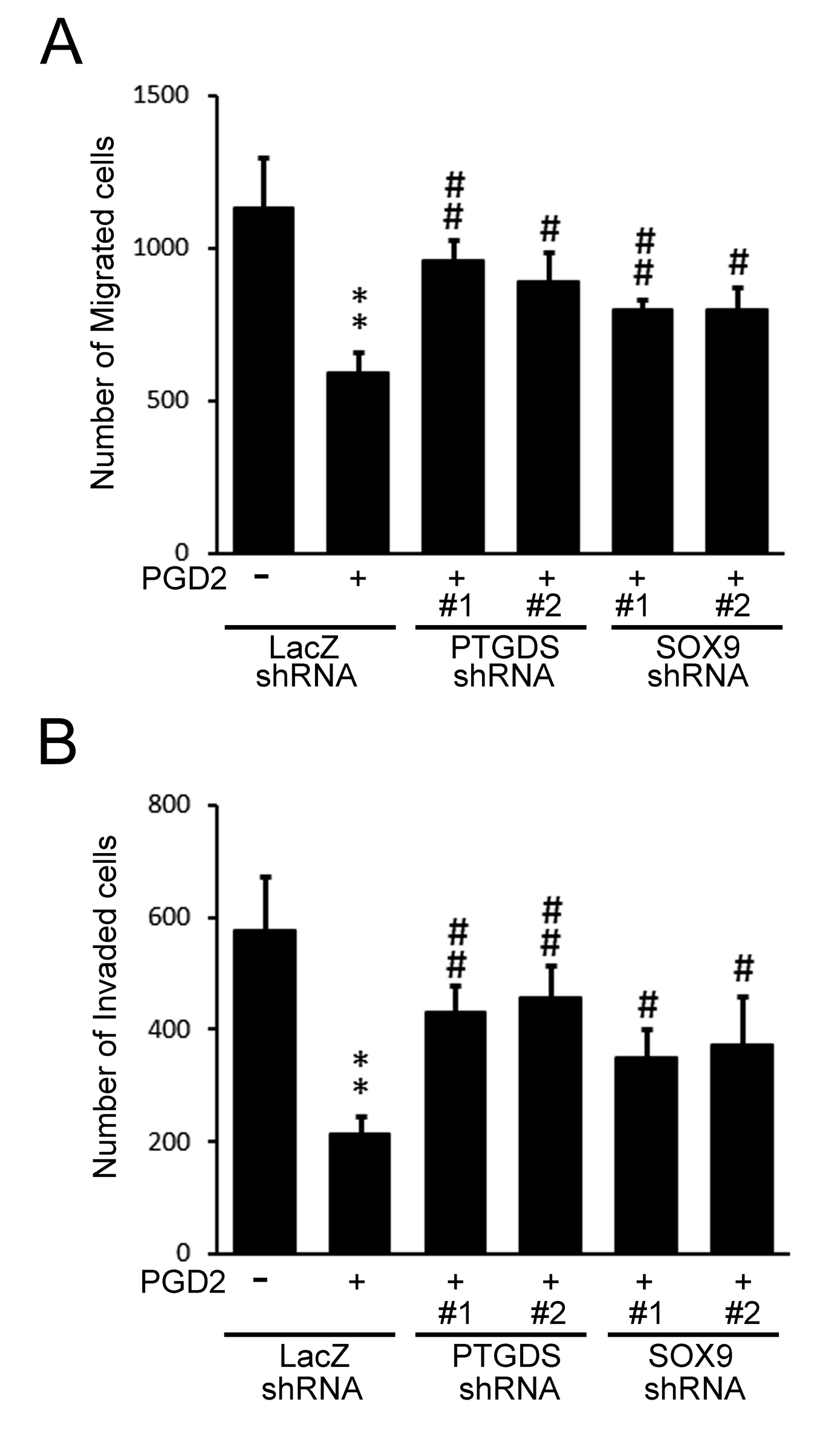

Supplement: Additional file 2: Figure S2 — PTGDS and SOX9 shRNAs alleviate PGD2-mediated suppression of cell migration and invasion. NT2/D1 cells were transduced with indicated shRNA for 72 h and then incubated with 500 ng/mL of PGD2 or ethanol vehicle for 24 h. Cells were subsequently prepared for analysis of cell migration (A) and invasion (B). Representative results of three independent experiments are shown. Student’s t-test: **, P < 0.01 versus control; #, P <0.01; ##, P <0.001 versus PGD2-treated cells that transduced with shLacZ. [file 1423-0127-20-30-S2.tiff]
